# Effectiveness and cost-effectiveness of sentinel lymph node biopsy compared with axillary node dissection in patients with early-stage breast cancer: a decision model analysis

**DOI:** 10.1038/bjc.2012.62

**Published:** 2012-03-13

**Authors:** H Verry, S J Lord, A Martin, G Gill, C K Lee, K Howard, N Wetzig, J Simes

**Affiliations:** 1 NHMRC Clinical Trials Centre, University of Sydney, Locked Bag 77, Camperdown, New South Wales 2050, Australia; 2 Department of Surgery, University of Adelaide, Adelaide, South Australia 5005, Australia; 3 School of Public Health, University of Sydney, Edward Ford Building A27, Camperdown, New South Wales 2006, Australia; 4 Wesley Medical Centre, Sandford Jackson Building, 30 Chasely Street, Brisbane, Queensland 4066, Australia

**Keywords:** cost, effectiveness, breast cancer, decision, model, sentinel node biopsy

## Abstract

**Background:**

Sentinel lymph node biopsy (SLNB) is less invasive than axillary lymph node dissection (ALND) for staging early breast cancer, and has a lower risk of arm lymphoedema and similar rates of locoregional recurrence up to 8 years. This study estimates the longer-term effectiveness and cost-effectiveness of SLNB.

**Methods:**

A Markov decision model was developed to estimate the incremental quality-adjusted life years (QALYs) and costs of an SLNB-based staging and management strategy compared with ALND over 20 years’ follow-up. The probability and quality-of-life weighting (utility) of outcomes were estimated from published data and population statistics. Costs were estimated from the perspective of the Australian health care system. The model was used to identify key factors affecting treatment decisions.

**Results:**

The SLNB was more effective and less costly than the ALND over 20 years, with 8 QALYs gained and $883 000 saved per 1000 patients. The SLNB was less effective when: SLNB false negative (FN) rate >13% 5-year incidence of axillary recurrence after an SLNB FN>19% risk of an SLNB-positive result >48% lymphoedema prevalence after ALND <14% or lymphoedema utility decrement <0.012.

**Conclusion:**

The long-term advantage of SLNB over ALND was modest and sensitive to variations in key assumptions, indicating a need for reliable information on lymphoedema incidence and disutility following SLNB. In addition to awaiting longer-term trial data, risk models to better identify patients at high risk of axillary metastasis will be valuable to inform decision-making.

Sentinel lymph node biopsy (SLNB) has been widely adopted as a less invasive surgical alternative to axillary lymph node dissection (ALND) for staging early breast cancer. When SLNB indicates axillary disease is not present, patients may be spared ALND surgery. This strategy reduces the risk of immediate complications and long-term arm morbidity, such as lymphoedema, for patients at low risk of nodal metastasis.

Over the last decade, clinical trials comparing the effectiveness of SLNB and ALND have randomised over 9000 patients with early breast cancer (Mansel *et al*, 2006; Veronesi *et al*, 2006; Zavagno *et al*, 2008; Canavese *et al*, 2009; Gill, 2009; Krag *et al*, 2010). They have demonstrated that SLNB reduces the risk of arm morbidity and lymphoedema, and shortens the postoperative hospital stay without significantly increasing locoregional recurrence or survival, with two trials reporting data at mean follow-up times of approximately 8 years (Krag *et al*, 2010; Veronesi *et al* 2010). Clinical guidelines recommend SLNB, by a team experienced in SLNB mapping and excision, as the standard of care for axillary staging for patients with clinically node-negative disease.

Despite the advantages of SLNB, unanswered questions remain. Sentinel lymph node biopsy carries a risk of false negative (FN) findings, with published estimates varying from 0–29% (meta-analysis summary estimate 7.0%, Kim *et al*, 2006). Longer follow-up data are needed to quantify the impact of FN findings on locoregional recurrence and survival, and the longer-term benefits of avoiding ALND, to help counsel patients. In particular, evidence on patient or tumour factors that define groups who may not be appropriate candidates for SLNB is lacking. For example, the benefit of SLNB for patients with large tumours remains unclear. Four of six published SLNB trials restricted eligibility to patients with tumours less than 2–3 cm (Veronesi *et al*, 2006; Zavagno *et al*, 2008; Canavese *et al*, 2009; Gill, 2009). Only two ongoing trials, NSABP 32 and ALMANAC, included patients with operable tumours of any size, with 20–25% of subjects having a tumour larger than 2 cm (Mansel *et al*, 2006; Krag *et al*, 2010).

Decision analysis synthesises the best available evidence in a systematic way to provide estimates of longer-term outcomes for different interventions over a range of clinical scenarios. This approach can be used to explicitly demonstrate how current trial evidence applies to different patient subgroups, and allows this evidence to be combined with subjective patient-rated preferences (utilities; Drummond *et al*, 2005) and the financial implications of treatment strategies.

The aim of this study was to develop a decision analytic model to estimate the 20-year effectiveness and cost-effectiveness of SLNB compared with ALND in the management of patients with early breast cancer. We explore the implications of extending SLNB to patients with a relatively high risk of lymph node metastasis, to provide estimates of efficacy where limited trial data exist. A secondary aim was to identify assumptions in the model, where little evidence was available, that were critical to conclusions, to highlight research priorities where more evidence is needed to inform decision-making.

## Methods

A decision model was developed to estimate the benefits, risks and costs of a staging and management strategy, based on SLNB compared with ALND over a 20-year time horizon in a simulated cohort of patients with early breast cancer and baseline characteristics similar to patients in the Australian Sentinel Node Axillary Clearance (SNAC) trial (Gill, 2009).

To define risk estimates for inclusion in the model, we reviewed six published, randomised controlled trials comparing SLNB and ALND that have reported on one or more relevant clinical endpoints—arm lymphoedema, axillary recurrence, overall survival at 12 months or longer for each staging strategy, together with information about SLNB performance (FN and failure rates) to allow an assessment of applicability to current practice standards (Mansel *et al*, 2006; Veronesi *et al*, 2006; Zavagno *et al*, 2008; Canavese *et al*, 2009; Gill, 2009; Krag *et al*, 2010). Costs were estimated from the perspective of the Australian health care system and included the direct health care costs associated with the 20-year natural history of breast cancer.

The net effectiveness of each strategy was quantified in terms of quality-adjusted life years (QALYs; Carr-Hill, 1989). This involves weighting the time spent in each health state by the health-related quality-of-life value (utility) associated with that state, where 0=death and 1=full health. Incremental cost-effectiveness was measured in terms of the cost per QALY gained. An annual discount rate of 5% was applied to all future costs and outcomes (Australian Government, 2011b).

We explored the impact of varying assumptions about both the costs and effects of each staging strategy within a plausible range for different clinical settings in a sensitivity analysis.

### Model structure

**Figure 1:**
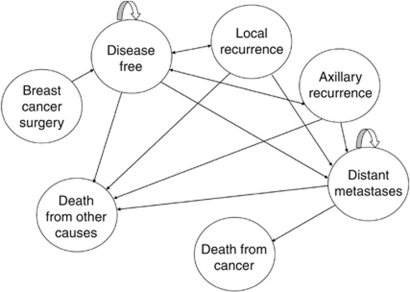
Health states following primary treatment for early breast cancer included in the decision model. All patients began the model in the disease-free state after their primary treatment; the likelihood of moving from one state to another at the end of a cycle was governed by a series of transition probabilities. After the first cycle, patients could remain disease free, or progress to local recurrence, axillary recurrence or distant metastases. Patients with a local or axillary recurrence could recover and return to disease free, or progress to a distant metastases. Once diagnosed with distant metastases, patients could either remain living with the metastases for the next cycle or else die from cancer. Death from cancer could only occur following distant metastases, whereas death from non-breast cancer-related causes could occur at any point in the model. Circular arrows indicate that patients can remain in this state.

The process defines a set of discrete health states following breast cancer and a set of probabilities that governs the likelihood of transitioning from one state to another at the end of each 1-year cycle (Drummond *et al*, 2005). The Markov process and health state transitions used are represented in Figure 1. Each health state was assigned an estimate of the cost required to provide appropriate health care over the cycle and a utility weighting that reflected the QALYs gained per cycle. A half-cycle correction was used in the Markov process.

Key assumptions about ALND and SLNB outcomes were: ALND provides only true positive or true negative results to guide appropriate management. Sentinel lymph node biopsy can also provide true positive or true negative results, but is also capable of generating a FN result (defined as a true finding of no metastases in the sentinel node and any accessory node in a patient who has occult metastases in other axillary nodes that have not been sampled), or failing to generate a result at all when the sentinel node cannot be identified. Patients with a true negative or FN SLNB finding are classified as having no axillary metastases and do not receive ALND; both positive SLNB cases and failed SLNB cases undergo ALND, consistent with current clinical guidelines.

### The SLNB performance characteristics, transition probabilities and health-state utilities used in model

All probabilities and health-state utilities used in the model are listed in Table 1, together with the ranges applied in the sensitivity analyses. The hypothetical patient cohort was assigned the average age (58 years), tumour grade (2) and risk of node-positive disease (26.9%) of the SNAC population (Gill, 2009). To explore potential implications of extending SLNB to a higher-risk population, we repeated our analyses for patients with a risk of lymph node involvement of 50%, reflecting the probability of lymph node involvement in patients with large or multifocal primary tumours (Laura *et al*, 2006; Behm and Buckingham, 2008; Ozmen *et al*, 2008).

Data from the SNAC trial was used to estimate rates of SLNB failure and SLNB FN findings for the base-case analysis. The risk of axillary recurrence for an FN SLNB was derived from the NSABP-32 trial data (the largest published trial, *n*=5611, Krag *et al*, 2010), by attributing the excess regional node recurrences for patients with a negative SLNB finding in the SLNB arm, compared with the ALND arm, entirely to the effect of FNs. This resulted in an estimated 7.7% of FN SLNB presenting with axillary recurrence within 5 years. The model was calibrated to reflect evidence that the risk of recurrence after primary treatment is highest in the first 5 years after diagnosis and diminishes progressively over time (Saphner *et al*, 1996).

The utility weights applied to the discrete health states were obtained from a breast cancer population using the standard gamble method (Kanis *et al*, 2003). As we did not find any published data on the utility of breast cancer patients with lymphoedema, a ‘disutility’ (quality-of-life decrement) was applied to patients with moderate-severe lymphoedema. This disutility was conservatively assumed to be 0.03 in the base-case analysis, which has been defined as the smallest clinically important difference in utility (Nichol and Epstein, 2008). A range of 0.01 to 0.05 was tested in the sensitivity analysis to reflect the uncertainty surrounding this estimate. The probability of experiencing moderate–severe arm lymphoedema following ALND or SLNB was estimated at 18% and 12%, respectively, based on the proportion of patients in the SNAC trial with an increase in arm volume of more than 15% after 3 years.

The per-annum risk of non-cancer death was derived from mortality data for women with a median age of 58 years (Australian Bureau Of Statistics, 2010).

### Resource use and costs used in model

The model took the perspective of the Australian health care system and included the direct health care costs associated with the 20-year natural history of breast cancer (Table 2).

The cost of the SLNB and ALND procedures were based on Australian Revised Diagnosis Related Group (AR-DRG) cost weights (Australian Government, 2008), adjusted to reflect length-of-stay data reported by SNAC (Gill, 2009). An additional cost was added to the inpatient cost of the SLNB procedure to include lymphoscintigraphy and lymphotropic dye injection costs (Australian Government, 2011a). The SNAC trial reported higher rates of postoperative seroma and infection following ALND than SLNB. It was assumed that the costs associated with these complications were accounted for in the longer hospital stay for ALND.

The initial surgery and adjuvant therapy costs were accrued before the first cycle of the Markov process. A cost was allocated to each health state on the basis of the resource usage level required to provide appropriate health care over each (1 year) cycle. A one-off transition cost was assigned to patients who died, or who entered the local recurrence or axillary recurrence states. Resource-use data for the long-term management of breast cancer were based on Australian clinical guidelines and include inpatient (AR-DRG), outpatient (medicare benefits schedule) and pharmaceutical (pharmaceutical benefits schedule) costs (Australian Government, 2008; Australian Government, 2011a, 2011b).

## Results

Using base-case assumptions, the model estimated that SLNB results in 1.1 more axillary recurrences per 1000 patients at 5 years, and 1.9 more axillary recurrences per 1000 patients at 20 years than ALND (Table 3). The estimated impact of this difference on the incidence of distant metastases and cancer death at 5 and 20 years are shown in Table 3. On the basis of these results, SLNB is estimated to be associated with a reduction of 10.4 life years per 1000 patients treated over 20 years. However, when taking into account the quality-of-life benefit gained by avoiding lymphoedema, SLNB was more effective than ALND, generating an additional 10.2 QALYs per 1000 patients over 20 years (8.2 QALYs after discounting future gains at 5% per annum; Table 3). Sentinel lymph node biopsy also saved AU$883 000 per 1000 patients over 20 years compared with ALND after discounting (Table 3).

Sentinel lymph node biopsy remained both more effective and less costly than ALND when all other parameters in the model were varied over their plausible range (Table 4).

**Figure 2:**
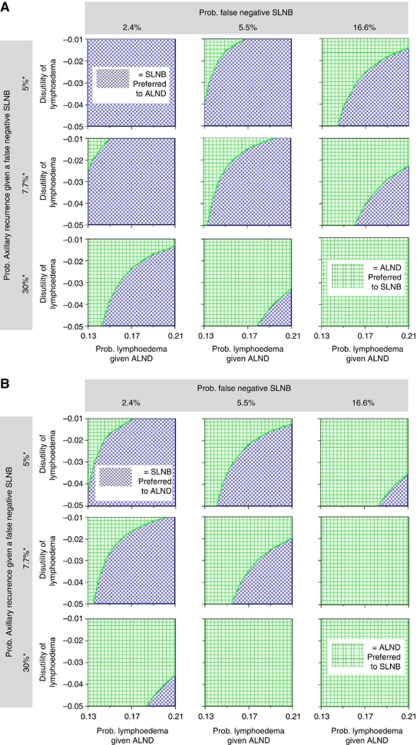
Multi-way sensitivity analysis. (**A**) Individuals with a 27% risk of axillary lymph node metastasis. The blue shading represents the set of probabilities and disutilities where SLNB leads to more QALYs gained, whereas the green represents the combination of probabilities and disutilities when ALND produces higher QALYs. ^*^Probability of axillary recurrence over initial 5 years of follow-up. (**B**) Individuals with a 50% risk of axillary lymph node metastasis. The blue shading represents the set of probabilities and disutilities where SLNB leads to more QALYs gained, whereas the green represents the combination of probabilities and disutilities when ALND produces higher QALYs. ^*^Probability of axillary recurrence over initial 5 years of follow-up. The colour reproduction of this figure is available at the *British Journal of Cancer* online.

A multiway sensitivity analysis examined the joint effects of uncertainty in the parameters for which an efficiency threshold was identified. Figure 2a shows the results of these parameters being simultaneously varied, assuming a population risk of nodal metastases of 27%. Each panel depicts a threshold plane shaded according to the preferred strategy (according to the QALYs generated) for a given combination of parameter estimates. The upper left-hand panel illustrates that SLNB (dark shading) was always preferred over ALND (light shading), irrespective of the estimated probability and disutility of lymphoedema, when the estimated risk of an FN SLNB and the risk of axillary recurrence, given an FN SLNB, were set to the lower end of the plausible range. The lower right-hand panel illustrates that this result was reversed, with ALND being preferred, when the estimated risk of an FN SLNB and the risk of axillary recurrence, given an FN SLNB, were set to the higher end. The panel in the centre illustrates that the preferred treatment was sensitive to the estimated probability and disutility of lymphoedema when the risk of an FN SLNB and the risk of axillary recurrence, given an FN SLNB, were set to the base-case estimates. When the multiway sensitivity analysis was repeated for a population with a 50% risk of nodal metastases, the results were more favourable towards ALND as shown in Figure 2b.

## Discussion

This modelled analysis indicates that SLNB is a more effective and marginally less costly staging strategy than ALND over a 20-year follow-up period. The base-case analysis for patients with a low risk of axillary metastases (e.g., a 60-year-old woman with a 2-cm tumour, histological grade 2 and non-palpable lymph nodes) demonstrated that the benefits of avoiding lymphoedema alone outweighed the potential small long-term risks associated with an FN SLNB rate of 5.5%, with only a modest cost saving of $883 over 20 years per patient.

Our analysis suggests SLNB remains more effective than ALND, even in settings where the risk of an FN result is as high as 13%. To put this threshold value in context, a systematic review of 69 SLNB accuracy studies conducted between 1970 and 2003 (total 7765 mapped sentinel nodes) found 81% of studies reported an SLNB FN rate lower than 13% (Kim *et al*, 2006). Current SLNB surgical training and quality control procedures aim to achieve FN rates of 5% or lower (Lyman *et al*, 2005).

We also explored the possible implications of extending SLNB to patients at higher risk of axillary metastasis where trial evidence is lacking (e.g., a 35-year-old woman with a 4-cm tumour, histological grade 3 and nonpalpable lymph nodes). On the basis of the current practice in which all patients with a positive SLNB proceed to ALND, our modelled analysis indicates ALND is the more effective strategy in this patient group (ALND favoured for patients with risk of axillary metastases >48%, Table 4).

Conclusion in favour of SLNB or ALND also depend on the true risk of lymphoedema associated with ALND compared with SLNB, the subjective impact to an individual on the symptoms of lymphoedema, and the range of uncertainty around the risk of axillary recurrence due to an FN SLNB. Sentinel lymph node biopsy became less effective than ALND if the risk of moderate–severe lymphoedema after ALND was set to 14%, an absolute risk difference of ⩽2%. Such a small difference is unlikely on the basis of current trial evidence of differences in lymphoedema and arm symptoms, including the NSABP-32 (5% difference in patient-reported moderate–severe arm swelling at 36 months, Land *et al*, 2010) and the ALMANAC trial (7% difference in the proportion of patients reporting substantial arm swelling at 18 months, Fleissig *et al*, 2005).

Exploring the uncertainty about the longer-term risk of axillary recurrence after an FN SLNB, we found the 5-year risk of axillary recurrence after an FN SLNB would need to exceed 19% to outweigh the benefits of avoiding lymphoedema, that is, at least 1 in 5 patients with an FN SLNB result experienced an axillary recurrence within 5 years. This probability cannot be directly measured, but recent clinical trials (Zavagno *et al*, 2008; Krag *et al*, 2010; Veronesi *et al*, 2010) and observational studies (Veronesi *et al*, 2009) provide data to estimate that the true risk is lower than this threshold, including the possibility of no increased risk. The estimate used for our base-case analysis was derived from 8-year data reported from NSABP B-32 (Krag *et al*, 2010). The low risk of axillary recurrence in this trial may be attributed to the use of adjuvant therapies; most patients (84%) with a negative SLNB in this trial received adjuvant endocrine and/or chemotherapy (Krag *et al*, 2010), and most receive radiotherapy to the breast and often the axillary tail of the breast with lower nodes included in those radiation fields. Recent results from the American College of Surgeons Oncology Group Z0011 trial provide further evidence that SLNB is not inferior to ALND in women with one to two positive sentinel nodes when combined with current adjuvant radiotherapy and chemotherapy treatments (Giuliano *et al*, 2011).

For patients with Stage I–IIa disease, representing the average trial patient, we found that even assuming a modest preference for avoiding lymphoedema (disutility of 0.03), the avoidance of arm morbidity through SLNB would outweigh these small potential risks of FN results. The disutility value of 0.03 has been defined as the smallest difference in utility that is considered to be clinically important (Nichol and Epstein, 2008), and in the context of our model, implies that patients would equate 10 years of life with moderate–severe lymphoedema to 9.7 years of life in full health. In contrast, for patients at high risk of axillary metastases, ALND was more effective than SLNB at a disutility for lymphoedema at 0.03. However, if patients more strongly preferred avoiding lymphoedema (e.g., disutility 0.04 or more), SLNB would also be more effective than ALND after taking into account the quality-of-life benefits of avoiding lymphoedema.

Our results will be helpful in counselling and individualising surgical treatment decisions for patients with early-stage breast cancer. Figure 2a and b provide a visual summary of findings from this study to inform these discussions between surgeons and patients. Overall, these results highlight the importance of patient preference in decision-making about SLNB, in particular in women at high risk of nodal metastases. For patients assessed at low-average risk of axillary metastases (Figure 2a), the centre box represents the woman at average risk with an SLNB FN rate fixed at 5.5%, based on the SNAC trial data (middle column) and the axillary recurrence rate estimated from trial data (middle row). It shows SLNB (dark shading) is favoured as producing more QALYs than ALND across a wide range of estimates for the probability of lymphoedema after ALND and the disutility of lymphoedema. In this scenario, ALND (light shading) would only be considered more favourable under extreme assumptions about the low probability of lymphoedema after ALND, or for patients with a very weak preference for avoiding lymphoedema. If shifting to a setting where the SLNB FN rate may be as high as 16.6% (as reported in the GIVOM trial), the middle box in the right-hand column shows ALND would be more favourable. However, conclusions would shift to favour SLNB with only moderate changes in estimates about lymphoedema risk and patient preferences.

Observational studies have indicated patients with tumours over 3 cm, multifocal tumours and tumours with lymphovascular involvement have a probability of axillary metastases of 50% and higher (Laura *et al*, 2006; Behm and Buckingham, 2008; Ozmen *et al*, 2008). Although awaiting trial direct evidence about the role of SLNB in these patient groups from ongoing trials, including the SNAC2 trial (Wetzig *et al*, 2011), Figure 2b illustrates relevant issues when discussing SLNB and ALND options in these patient groups.

Costs vary between different countries; however, the two major factors driving differences in costs between SLNB and ALND – the longer hospital stay and higher risk of lymphoedema associated with ALND – are likely to be consistent internationally. The SLNB-based strategy was more expensive than ALND when the cost difference between the two procedures was less than AU$741 per patient. We assumed 79% of SLNB-positive procedures incurred a second operation for ALND at a later date (Gill, 2009). The cost-effectiveness of SLNB would be even more favourable if it were possible to identify and manage SLNB-positive cases at the time of the initial operation, avoiding reoperation and a second hospital admission. Another driving factor for the size of difference in both the costs and effects between these strategies is the proportion of patients offered SLNB, who test positive and proceed to ALND, incurring the extra costs and associated morbidity for the second procedure.

This costing does not include the costs borne by the patient and society. In particular, the modelled costs of lymphoedema are likely to underestimate the costs to the patient in Australia where access to publicly funded physiotherapy and occupational therapy services for lymphoedema are limited. The major strength of this study is to provide a framework that combines trial evidence of clinical efficacy and patient preference in individualising surgical treatment decision for patients with early breast cancer. This modelling approach provides the unique advantage to assess the relative long-term morbidities of the two surgical approaches as tradeoffs between improved quality-of-life and potential small survival reduction for SLNB compared with ALND. It also provides an evidence-based estimate of the relative long-term benefits of SLNB over ALND and the potential impact of extending SLNB to patient groups where limited evidence is available from clinical trials.

The main limitation is that the model does not capture all possible consequences of SLNB and ALND. For example, the benefits of SLNB are limited to an estimation of improved quality-of-life associated with avoiding arm lymphoedema, and the potential harms associated with SLNB due to patient anxiety about the need to proceed to ALND and delay in definitive surgery are not explored. The model does not attempt to reflect the entire complexity of practice by mapping out all possible breast cancer outcomes or treatment strategies. Nevertheless, we believe the model structure reflects the major sources of differences between SLNB and ALND that influence long-term cost-effectiveness.

Overall, our finding that a staging strategy using SLNB is both more effective but marginally less costly than ALND over 20 years is not unexpected, and is consistent with current clinical guidelines (Aebi *et al*, 2010; NCCN, 2011). More intriguing is our finding that this advantage, 0.0082 QALYs and $883 saved per patient, appeared to be smaller than the perceived benefits based on clinical experience and enthusiasm, and consumer demand. Possible reasons for this discrepancy are: first, that the model estimate of the patient disutility associated with lymphoedema does not fully capture the difference in all patient-important outcomes such as short- and long-term morbidity and the broader social and financial implications of these outcomes for patients; second, it is possible that the population benefits of SLNB are more modest than generally perceived.

Unfortunately, current trials have used different definitions of lymphoedema, which limits comparisons and pooling of data to better estimate the benefits of avoiding ALND. There is a need for a standard clinical definition and criteria for the measurement of arm lymphoedema for future trial analyses. There is also no commonly accepted measure of ALND-related quality-of-life. SLNB trials have each used different validated measurement tools to report statistically significant health-related quality-of-life advantages for patients receiving SLNB compared with ALND at up to 3 years, although the difference in quality-of-life scores appears to reduce over this time period, with two trials reporting no significant difference in the overall quality-of-life scores at 2 (Del Bianco *et al*, 2008) and 3 years (Land *et al*, 2010). It would be very valuable if these data were used to derive a more direct estimate of difference in utility, the measure used to calculate QALYs, between the two staging strategies for use in decision models such as ours. Studies to measure the effects of arm lymphoedema on costs to patient, health system and society are also needed for more precise estimates of cost-effectiveness.

In conclusion, this model identifies factors that influence SLNB effectiveness and cost-effectiveness, and hence, are important to consider when individualising treatment and counselling patients, as well as identifying areas where more reliable estimates will be of value. Although the modelled analysis indicates that SLNB is a cost-effective treatment over 20 years, this does not replace the need for empirical evidence. Information about the long-term incidence and disutility of lymphoedema is needed, and will be particularly helpful to inform decisions for patients at high risk of axillary metastases. If the quality-of-life benefits of SLNB are found to be smaller than our modelled estimates, then long-term trial data will be critical to better estimate or exclude small differences in cancer outcomes. Well-validated prognostic models will also be valuable to better select patients for SLNB, who are at low risk of axillary metastasis or can safely avoid ALND if SLNB is positive.

## Figures and Tables

**Table 1:** SLNB characteristics, transition probabilities and health-state utilities

		**Range**		
**Variable**	**Base case**	**Low**	**High**	**Source**
*Proportions*				
Node positive patients^a^	0.269	0.2	0.46	Gill (2009)
SLNB procedures failing	0.06	0.001	0.071	Gill (2009)
FN SLNB result	0.055	0.024	0.166	Gill (2009)
ALND patients experiencing lymphoedema	0.176	0.1312	0.2112	SNAC trial data
SLNB patients experiencing lymphoedema	0.119	0.0416	0.1428	SNAC trial data
				
*Annual probabilities*				
Local (ipsilateral) recurrence as first event^b^	0.0055	0.0044	0.0066	Krag *et al* (2010)
Axillary recurrence as first event^b^	0.0008	0.0006	0.0010	Krag *et al* (2010)
Given FN^b^	0.0160	0.0102	0.0689^d^	Krag *et al* (2010)
Distant metastases				
As a first event, node positive	0.0126	0.0109	0.0144	de Bock *et al* (2009)
As a first event, node negative	0.0063	0.005	0.0075	Veronesi *et al* (2009)
After local recurrence, node positive	0.1246	0.0997	0.1496	Wapnir *et al* (2005)
After local recurrence, node negative	0.0772	0.0429	0.0927	Anderson *et al* (2009)
After axillary recurrence, node positive	0.2063	0.1305	0.2759	Wapnir *et al* (2005)
After axillary recurrence, node negative	0.2259	0.1674	0.2834	Anderson *et al* (2009)
Death following distant mets	0.2970	0.2729	0.3313	Gennari *et al* (2005)
Death from other causes	Changes over time	—	—	ABS mortality data^c^
				
*Utilities*				
Disease free	0.989	0.79	1	Karnon *et al* (2008)
Axillary recurrence	0.911	0.73	1	Karnon *et al* (2008)
Local recurrence	0.911	0.73	1	Karnon *et al* (2008)
Distant metastases	0.796	0.64	0.96	Karnon *et al* (2008)
Lymphoedema penalty	−0.03	−0.05	−0.01	Assumption

Abbreviations: ALND=axillary lymph node dissection; CI=confidence interval; FN=false negative; SNAC=sentinel node axillary clearance; SNLD=sentinel lymph node biopsy.

a Sensitivity analysis reflects low 95% CI from SNAC, as well as upper CI from other randomised controlled trials.

b Probability halves after the first 5 years, and halves again after 10 years.

c General mortality rate for females aged 55–74, adjusted to exclude breast cancer deaths.

d Upper sensitivity estimate is conservatively based on an assumption that 30% of FNs will present with axillary recurrence after 5 years.

**Table 2:** Resource use and costs

**Costs^a^**	**Source**	**Unit cost ($)**	**Total cost ($)**
*Treatment*			
ALND procedure	AR-DRG	—	5576.45
SLNB-negative procedure	AR-DRG, MBS	—	4206.38
SLNB positive, ALND following	AR-DRG, MBS	—	7771.28
SLNB fail, ALND following	AR-DRG, MBS	—	5576.45
			
*Adjuvant therapy* b			
Radiotherapy (86% of ALND, 89% of SLNB)^c^	MBS	—	5130.40
First-line chemotherapy (30% for ALND, 31% for SLNB)^d^	MBS,PBS	—	16 160.43
Endocrine therapy – 5 years (81%)	PBS	—	10 960.95
Herceptin therapy – 1 year (29%)	Cancer Institute, MBS	—	64 032.80
			
*Follow-up schedule (annual)*	—	—	254.10
History and examination × 2	MBS	82.30	—
Mammography × 1	MBS	89.50	—
			
*Lymphoedema (annual)*	—	—	1198.60
Lymphoedema mobility clinic	AR-DRG	959.00	—
Outpatient physiotherapy clinic × 4	MBS	59.90	—
			
*Local recurrence*	—	—	7658.40
Inpatient major surgical procedure	AR-DRG	6393.00	—
Radiotherapy (13%)^c^	MBS	5171.75	—
Specialist visits × 4	MBS	82.30	—
GP visits × 4	MBS	67.65	—
			
*Axillary recurrence*	—	—	24 555.97
Inpatient major surgical procedure	AR-DRG	6393.00	—
Radiotherapy (13%)^c^	MBS	5171.75	—
First-line chemotherapy (69%)^d^	MBS, PBS	16 160.43	—
Second-line chemotherapy (31%)^d^	MBS, PBS	18 664.31	—
Specialist visits × 4	MBS	82.30	—
GP visits × 4	MBS	67.65	—
			
*Distant metastases (annual)*	—	—	24 340.11
Inpatient procedure × 3 (70%)	AR-DRG	5190.33	—
Third-generation chemotherapy (100%)^d^	MBS, PBS	5419.06	—
Fourth-generation chemotherapy (50%)^d^	MBS, PBS	15 172.31	—
Specialist visits × 2	MBS	82.30	—
GP visits × 4	MBS	67.65	—
			
*End of life costs*	—	—	29 615.97
Equivalent to 2 × the initial stage of breast cancer	Baker *et al* (1991)	—	—
			
*Death from other causes*	Seshamani and Gray (2005) JHE	—	8659.10

Abbreviations: ALND=axillary lymph node dissection; AR-DRG=Australian refined diagnostic related groups; MBS=medicare benefits schedule; PBS=pharmaceutical benefits schedule; SNAC=sentinel node axillary clearance; SNLD=sentinel lymph node biopsy.

a All costs are varied by 20% in the sensitivity analysis.

b The proportion of patients receiving each therapy is taken from SNAC.

c Radiotherapy costs include an initial and follow-up consultation, computed tomography planning and megavoltage – three fields (breast, boost and axilla) for 6 weeks.

d Chemotherapy costs include an initial and follow-up consultation, chemotherapy drug(s), drug administration and ancillary medications.

**Table 3:** Modelled estimates of outcomes for SLNB and ALND at 5 and 20 years

	**After 5 years**			**After 20 years**		
	**SLNB**	**ALND**	**Difference**	**SLNB**	**ALND**	**Difference**
*Cumulative incidences (per 1000)*						
Axillary recurrence	4.9	3.8	1.1	9.6	7.7	1.9
Distant metastases	44.5	44.2	0.3	174.2	172.8	1.4
Death from cancer	18.9	18.8	0.1	145.5	144.3	1.2
Death from other	18.4	18.4	0.0	159.8	160.1	−0.3
Years disease free	4822.50	4823.96	1.5	17 008.65	17 029.40	−20.75
Total life years	4926.88	4926.98	0.1	17 571.52	17 581.93	−10.41
						
*Cost effectiveness (per 1000)*						
QALYs discounted	4295.97	4290.92	5.05	11 302.40	11 294.20	8.20
Cost discounted ($AU millions)	46.04	46.66	−0.62	57.49	58.38	−0.88
Result when discounted	Favours SLNB^a^			Favours SLNB^a^		
						
QALYs undiscounted	4835.7	4830.0	5.7	17 205.2	17 195.0	10.2
Cost undiscounted ($AU millions)	46.63	47.27	−0.64	67.01	68.13	−1.12
Result when undiscounted	Favours SLNB^a^			Favours SLNB^a^		

Abbreviations: ALND=axillary lymph node dissection; QALY=quality adjusted life year; SLNB=sentinel node biopsy.

a Base case estimates indicated that SLNB was both cheaper and more effective than ALND.

**Table 4:** Results of one-way sensitivity analyses for parameters with cost or effectiveness thresholds

**Parameter description**	**Threshold value**
*Parameters with effectiveness thresholds*	
Probability of lymph node positive disease (%)	>48
Risk of axillary recurrence given FN SLNB (%)	>19
Probability of FN SLNB (%)	>13
Probability of lymphoedema in contral arm (i.e., ALND; %)	<14
Disutility of lymphoedema	<0.0124
	
*Parameters with cost thresholds*	
Cost SLNB positive^a^ ($)	>57 183
Cost SLNB negative^a^ ($)	>46 864
Cost ALND^a^ ($)	<46 679
Probability lymph node positive disease (%)	>43

Abbreviations: ALND=axillary lymph node dissection; FN, false negative; SLNB=sentinel node biopsy.

a Cost includes both surgery and adjuvant therapy

## References

[bib1] Aebi S, Davidson T, Gruber G, Castiglione M (2010) Primary breast cancer: ESMO Clinical Practice Guidelines for diagnosis, treatment and follow-up. Ann Oncol 21(Supplement 5): v9–v14 20555111 10.1093/annonc/mdq159

[bib2] Anderson SJ, Wapnir I, Dignam JJ, Fisher B, Mamounas EP, Jeong J, Geyer Jr CE, Wickerham DL, Costantino JP, Wolmark N (2009) Prognosis after ipsilateral breast tumor recurrence and locoregional recurrences in patients treated by breast-conserving therapy in five National Surgical Adjuvant Breast and Bowel Project protocols of node-negative breast cancer. J Clin Oncol 27(15): 2466–2473 19349544 10.1200/JCO.2008.19.8424 PMC2684852

[bib3] Australian Bureau of Statistics (ABS) (2010) Life tables, Australia, 2007–2009, cat. no. 3302.0.55.001. 8-12-2010

[bib4] Australian Government (2008) Australian Refined Diagnostic Related Groups (AR-DRG) Version 5.1. Commonwealth of Australia. 11-7-2008. Ageing DoHa

[bib5] Australian Government (2011a) Medicare Benefits Schedule. Commonwealth of Australia

[bib6] Australian Government (2011b) Schedule of Pharmaceutical Benefits. Commonwealth of Australia. 1-2-2011b

[bib7] Baker M, Kessler LG, Urban N, Smucker RC (1991) Estimating the treatment costs of breast and lung cancer. Med Care 29(1): 40–49 1986176 10.1097/00005650-199101000-00004

[bib8] Behm EC, Buckingham JM (2008) Sentinel Node Biopsy in Larger or Multifocal Breast Cancers: To Do or Not To Do. ANZ J Surg 78: 151–157 18269478 10.1111/j.1445-2197.2007.04392.x

[bib9] Canavese G, Catturich A, Vecchio C, Tomei D, Gipponi M, Villa G, Carli F, Bruzzi P, Dozin B (2009) Sentinel node biopsy compared with complete axillary dissection for staging early breast cancer with clinically negative lymph nodes: results of randomized trial. Ann Oncol 20(6): 1001–1007 19174453 10.1093/annonc/mdn746

[bib10] Carr-Hill RA (1989) Assumptions of the QALY Procedure. Soc Sci Med 29(3): 469–477 2762872 10.1016/0277-9536(89)90296-7

[bib11] de Bock GH, Putter H, Bonnema J, Van Der Hage JA, Bartelink H, Van De Velde CJ (2009) The impact of loco-regional recurrences on metastatic progression in early-stage breast cancer: a multistate model. Breast Cancer Res Treat 117(2): 401–408 19148746 10.1007/s10549-008-0300-2

[bib12] Del Bianco P, Zavagno G, Burelli P, Scalco G, Barutta L, Carraro P, Pietrarota P, Meneghini G, Morbin T, Tacchetti G, Pecoraro P, Belardinelli V, De Salvo GL (2008) Morbidity comparison of sentinel lymph node biopsy *versus* conventional axillary lymph node dissection for breast cancer patients: results of the sentinella-GIVOM Italian randomised clinical trial. Eur J Surg Oncol 35: 508 10.1016/j.ejso.2007.05.017 17614245

[bib13] Drummond MF, Sculpher MJ, Torrance GW, O’Brien BJ, Stoddart GL (2005) Methods for the Economic Evaluation of Health Care Programmes. Oxford University Press Inc.: New York

[bib14] Fleissig A, Fallowfield L, Langridge C, Johnson L, Newcombe RG, Dixon JM, Kissin M, Mansel RE (2006) Post-operative arm morbidity and quality of life. Results of the ALMANAC randomised trial comparing sentinel node biopsy with standard axillary treatment in the management of patients with early breast cancer. Breast Cancer Res Treat 95: 279–293 16163445 10.1007/s10549-005-9025-7

[bib15] Gennari A, Conte P, Rosso R, Orlandini C, Bruzzi P (2005) Survival of metastatic breast carcinoma patients over a 20-year period: a retrospective analysis based on individual patient data from six consecutive studies. Cancer 104(8): 1742–1750 16149088 10.1002/cncr.21359

[bib16] Gill G (2009) Sentinel-lymph-node-based management or routine axillary clearance? One-year outcomes of sentinel node biopsy *versus* axillary clearance (SNAC): a randomized controlled surgical trial. Ann Surg Oncol 16(2): 266–275 19050973 10.1245/s10434-008-0229-z

[bib17] Giuliano AE, Hunt KK, Ballman KV, Beitsch PD, Whitworth PW, Blumencranz PW, Leitch AM, Saha S, McCall LM, Morrow M (2011) Axillary dissection *vs* no axillary dissection in women with invasive breast cancer and sentinel node metastasis. JAM A 305(6): 569–575 10.1001/jama.2011.90 PMC5389857 21304082

[bib18] Kanis JA, Brazier JE, Stevenson M, Calvert N, Lloyd Jones M (2003) Treatment of established osteoporosis: a systematic review and cost-utility analysis. Health Technol Assess 6(29): 1–146 10.3310/hta6290 12654239

[bib19] Karnon J, Delea T, Barghout V (2008) Cost utility analysis of early adjuvant letrozole or anastrozole versus tamoxifen in postmenopausal women with early invasive breast cancer: the UK perspective. Eur J Health Econ 9(2): 171–183 17602251 10.1007/s10198-007-0058-1

[bib20] Kim T, Giuliano AE, Lyman GH (2006) Lymphatic mapping and sentinel lymph node biopsy in early-stage breast carcinoma: a metaanalysis. Cancer 106(1): 4–16 16329134 10.1002/cncr.21568

[bib21] Krag DN, Anderson SJ, Julian TB, Brown AM, Harlow SP, Costantino JP, Ashikaga T, Weaver DL, Mamounas EP, Jalovec LM, Frazier TG, Noyes RD, Robidoux A, Scarth HM, Wolmark N (2010) Sentinel-lymph-node resection compared with conventional axillary-lymph-node dissection in clinically node-negative patients with breast cancer: overall survival findings from the NSABP B-32 randomised phase 3 trial. Lancet Oncol 11(10): 927–933 20863759 10.1016/S1470-2045(10)70207-2 PMC3041644

[bib22] Land SR, Kopec JA, Julian TB, Brown AM, Anderson SJ, Krag DN, Christian NJ, Costantino JP, Wolmark N, Ganz PA (2010) Patient-reported outcomes in sentinel node-negative adjuvant breast cancer patients receiving sentinel-node biopsy or axillary dissection: National Surgical Adjuvant Breast and Bowel Project phase III protocol B-32. J Clin Oncol 28(25): 3929–3936 20679600 10.1200/JCO.2010.28.2491 PMC2940391

[bib23] Laura S, Coombs NJ, Ung O, Boyages J (2006) Tumour size as a predictor of axillary node metastases in patients with breast cancer. ANZ J Surg 76: 1002–1006 17054550 10.1111/j.1445-2197.2006.03918.x

[bib24] Lyman GH, Giuliano AE, Somerfield MR, Benson III AB, Bodurka DC, Burstein HJ, Cochran AJ, Cody III HS, Edge SB, Galper S, Hayman JA, Kim TY, Perkins CL, Podoloff, Sivasubramaniam VH, Turner RR, Wahl R, Weaver DL, Wolff AC, Winer EP (2005) American Society of Clinical Oncology Guideline Recommendations for Sentinel Lymph Node Biopsy in Early Stage Breast Cancer. J Clin Oncol 23(30): 7703–7720 16157938 10.1200/JCO.2005.08.001

[bib25] Mansel RE, Fallowfield L, Kissin M, Goyal A, Newcombe RG, Dixon JM, Yiangou C, Horgan K, Bundred N, Monypenny I, England D, Sibbering M, Abdullah TI, Barr L, Chetty U, Sinnett DH, Fleissig A, Clarke D, Ell PJ (2006) Randomized multicenter trial of sentinel node biopsy *versus* standard axillary treatment in operable breast cancer: the ALMANAC Trial.(Erratum appears in J Natl Cancer Inst. 2006 Jun 21;98(12):876). J Natl Cancer Inst 98(9): 599–609 16670385 10.1093/jnci/djj158

[bib26] National Comprehensive Cancer Network, (2011) The NCCN Clinical Practice Guidelines in Oncology: Breast Cancer. Version 2.2011. 25-3-2011.

[bib27] Nichol M, Epstein J (2008) Separating gains and losses in health when calculating the minimum important difference for mapped utility measures. Qual Life Res 17(6): 955–961 18615271 10.1007/s11136-008-9369-7

[bib28] Ozmen V, Karanlik H, Cabioglu N, Igci A, Kecer M, Asoglu O, Tuzlali S, Mudun A (2008) Factors predicting the sentinel and non-sentinel lymph node metastases in breast cancer. Breast Cancer Res Treat 95: 1–6 10.1007/s10549-005-9007-9 16322900

[bib29] Saphner T, Tormey D, Gray R (1996) Annual Hazaed Rates of Recurrence for Breast Cancer After Primary Therapy. J Clin Oncol 14(10): 2738–2746 8874335 10.1200/JCO.1996.14.10.2738

[bib30] Seshamani M, Gray AM (2005) A longitudinal study of the effects of age and time to death on hospital costs. J Health Econ 23(2): 217–235 10.1016/j.jhealeco.2003.08.004 15019753

[bib31] Veronesi U, Galimberti V, Paganelli G, Maisonneuve P, Viale G, Orecchia R, Luini A, Intra M, Veronesi P, Caldarella P, Renne G, Rotmensz N, Sangalli C, Lima L, Tullii M, Zurrida S (2009) Axillary metastases in breast cancer patients with negative sentinel nodes: A follow up of 3548 cases. Eur J Cancer 45: 1381–1388 19128957 10.1016/j.ejca.2008.11.041

[bib32] Veronesi U, Paganelli G, Viale G, Luini A, Zurrida S, Galimberti V, Intra M, Veronesi P, Maisonneuve P, Gatti G, Mazzarol G, De CC, Manfredi G, Fernandez JR (2006) Sentinel-lymph-node biopsy as a staging procedure in breast cancer: update of a randomised controlled study. Lancet Oncol 7(12): 983–990 17138219 10.1016/S1470-2045(06)70947-0

[bib33] Veronesi U, Viale G, Paganelli G, Zurrida S, Luin A, Galimberti V, Veronesi P, Intra M, Maisonneuve P, Zucca F, Gatti G, Mazzarol G, De Cicco C, Vezzoli D (2010) Sentinel Lymph Node Biopsy in Breast Cancer: Ten-Year Results of a Randomised Controlled Study. Ann Surg 251(4): 595–600 20195151 10.1097/SLA.0b013e3181c0e92a

[bib34] Wapnir IL, Anderson SJ, Mamounas EP, Geyer Jr CE, Jeong J, Tan-Chiu E, Fisher B, Wolmark N (2005) Prognosis after ipsilateral breast tumor recurrence and locoregional recurrences in five National Surgical Adjuvant Breast and Bowel Project node-positive adjuvant breast cancer trials. J Clin Oncol 24(13): 2028–2037 10.1200/JCO.2005.04.3273 16648502

[bib35] Wetzig N, Campbell I, Gill G, Ung O, Collins J, Oliver D (2011) SNAC2: Sentinel node biopsy *versus* axillary clearance in women with operable breast cancer. ACTRN12605000409673

[bib36] Zavagno G, De Salvo GL, Scalco G, Bozza F, Barutta L, Del BP, Renier M, Racano C, Carraro P, Nitti D, GIVOM T (2008) A Randomized clinical trial on sentinel lymph node biopsy *versus* axillary lymph node dissection in breast cancer: results of the Sentinella/GIVOM trial. Ann Surg 247(2): 207–213 18216523 10.1097/SLA.0b013e31812e6a73

